# Supplementary material for: GC Content and Thermal Stability of Double-Stranded RNA: Fragments of Microsporidia Vairimorpha ceranae and Nosema bombycis AT-Rich Genes Are Sensitive to Standard Heat Treatment
Source: Int J Mol Sci. 2025 Oct 22;26(21):10270. doi: 10.3390/ijms262110270 (PMC12609917; doi:10.3390/ijms262110270)
Supplement: Supplementary file 1 [file ijms-26-10270-s001.zip › Figure S1. Sequences of control gene fragments with normal GC content.pdf]

M. persicae nuc (in pRSETRNAi 1 [40]), 679 bp, G/C 53.6%, 15 bp and longer regions without adjacent G or C 9.6%

GGGAGACCACAACGGTTTCCCTCTAGAAATAATTTTGTTTAACTTTAAGAAGGAGATATACATATG***GGATCC***TCCGACGCGTTGGT
CACGTGCGTCGGCGGCGACGTGCTCGCGTACCGAGGCCAGACGTACCGGTACGCCGCCTTCCAGTGCGACGGCATGCCCAA
GTCGGAGCTCCGCGTCACCGACGAAGTGTGCCAGCCGGCCAACTACACCGTGGCCGTGGTCGGTTTCCGGACGGATCGCGC
GTTCCTCCGGCTGTACGGCATGTGCTTCGACAAGTCGACCAAGAACAGCCTGTACACTTGGTACGACGCCCGGTCGCCGTACT
ACGACAACCACCAGAAGTATAGCAAGCGACCGTCGTTCAACAAAACCAAAGAGCTGTACGGCAACACGGACGTGAACAAGAAAT
ACACTTTCAAAGAACAGAGAAACACGGTGGCGACAATACTCAGATCGGACGAACTTGCGGATAAGTACATAAGGAATGATAATAA
ACACTTCCTGTCCCGGGGCCATTACGCAGCCAAAGTTGACTTTTTCTTTGCTTATGAACAAACAGCAACGTTTTACTATGCGAAC
GTTGCCCCGCAGTGGCAGATCTTCAACGGTGACATGTGGGCCGATCTGGAATCGACTACCCGGTCA***AAGCTT***GATCCGGCTGC
TAACAAAGCCC

L. decemlineata b'-COP (in pRSETRNAi 1 [40]), 401 bp, G/C 43.4%, 15 bp and longer regions without adjacent G or C 29.4%

GGGAGACCACAACGGTTTCCCTCTAGAAATAATTTTGTTTAACTTTAAGAAGGAGATATACATATG***GGATCC***GAGCTCGAGATCTG
CAGCTGGTACCATG***GAATTC***ATTCCTTTGAGGCACATTCCGACTATGTGAGATGTATAATAGTTCATCCAACCCAACCTTATATTCT
GACAAGTAGTGATGACATGCTCATCAAATTGTGGAATTGGGATAAGGCTTGGGCTTGTCAGCAGGTCTTTGAAGGCCACTCTCAT
TATATCATGCAAATCGCCATCAATCCAAAAGACAATAACACATTTGCGAGTGCTTCACTGGACCGTACTTTGAAAGTGTGGCAACT
TGGAGCATCAACAGCGAACTTCACCCTTG***AAGCTT***GATCCGGCTGCTAACAAAGCCC

L. decemlineata V-ATPase A - mov34 – actin (in pRSETRNAi 1 [40]), 869 bp, G/C 47.6%, 15 bp and longer regions without adjacent G and C 7.8%

GGGAGACCACAACGGTTTCCCTCTAGAAATAATTTTGTTTAACTTTAAGAAGGAGATATACATATG***GGATCC***CAACTTGATGTTGAT
TGGGTTGAATTCCCATTTGATAGTTCTGGAGAGTGAAGGCACGTTCACACCCTTGGGAATGTAGATACTTTCCGTCATGTCACAG
ATGTCTTTCAGCGGACGTTGGATACCATCAAAGATGGAACCCATAATACCTGGCCCAAGTTCCACAGATAGAGGTTTACCGGTAC
GCAACACAGGATCTCCCACCGTTACTCCAGAAGTTTCTTCATATACCTG***CTCGAG***CCAACTGTGGTATCTTTGATATCCCTCAGAA
GGTGCTCCACTCCCACCTCCTCTGCTTCTTCCGCCCCTATTTCGCTAGGTACATGCTCAAATGTTTTGGAAGTGGGCGATCCATC
ATCGTGGACTTCCTCCACAGCTTGATATGCCTCGGTTGGGAGGCCTAGATCCTTGGGTTTGGCATCAATTATTACCGACACAGAA
TTGGGACAGTATCTCCTAATTAGTTCGTTTATTGCTATGTCATTCTGGTCTAATTTTGGACCTGTGT***GGTACC***TCCAGAGAGGACG
GTGTTGGCGTACAAGTCCTTACGGATATCAACGTCACACTTCATGATGGAGTTGTAGACGGTTTCGTGGATACCGCTAGATTCCAT
ACCCAAGAAGGAAGGTTGGAACAAGGCTTCTGGGCAACGGAACCTCTCGTTACCAATGGTGATGACCTGTCCATCGGGAAGTT
CGTAGCTCTTTTCGAGGGAGGTTGAAGCAGCAGCGGTGGCCATTTCCTGTTCGAAGTCGAGGGCAACATAGCA***AAGCTT***GATC
CGGCTGCTAACAAAGCCC

V. ceranae helicase chimeric (in pRSETRNAi 2 [45]), 509 bp, G/C 41.3%, 15 bp and longer regions without adjacent G and C 22.4%

GGATCCAACACATACAAGACTTAGAGAATAATGCCTACTGGTGGCGGAAAATAAAGGCGTATGGTTCTAGAAAATTCAGCCCAC
TACTTTCACTGGTACATGAACTCCTGAATCCTTATGTGCCAGGGGCATGACTTTAGGCCGGATTATATGGCCGGATTATATTGAA
ATGAAAAAGTGCCTATAGTAGCACTTACAGCTACAATGTATCCATCAGTGCCTATAGGTAGCACTTACAGCTACCGCTACACCAA
ACACGATCAGTAGAGCTAGACATAGTCAAATGCGAAATGATCTCCGATAAATTAAAAAGAACGGATGTCTATACAAAGTGTAGCT
ACAATAGCCTTTGGTATGGGTATAGCCTTTGGTATGGGTATTGATAAGAAGACGTTAGATTCGTTATTCACAAAGTCTAGAAGGA
TACTACCAGACAGAGACAACGTGAAGAACTTAGGGAAAGTAGAACGAATTGTTACTC***AAGCTT***GATCCGGCTGCTAACAAAGCC
C

Figure 1S. Sequences of control gene fragments with normal GC content used for dsRNA synthesis. Regions without adjacent G and C are marked as grey boxes, cloning sites of restriction enzymes are in bold and italic, additional vector-derived sequences are underlined.
