# Supplementary material for: GC Content and Thermal Stability of Double-Stranded RNA: Fragments of Microsporidia Vairimorpha ceranae and Nosema bombycis AT-Rich Genes Are Sensitive to Standard Heat Treatment
Source: Int J Mol Sci. 2025 Oct 22;26(21):10270. doi: 10.3390/ijms262110270 (PMC12609917; doi:10.3390/ijms262110270)
Supplement: Supplementary file 1 [file ijms-26-10270-s001.zip › Figure S2. Sequences of N. bombycis fragments.pdf]

N. bombycis delta DNA pol. (in pRSETRNAi 2 [45]), 561 bp, G/C 37.6%, 15 bp and longer regions without adjacent G or C 36.4%

GGATCCTAAGAGAGGGTTTTATAGTCAACCGATTGCTGTTCTAGATTTCTCTTCTCTTTATCCTTCGATTATGATTAGTCGGAACTT
GTGTTATACAACTTTACTGACTAAGAAACAATATGAGAAGTACGGAGGAATCAAGTCACCAACTAATGATTACTTTGCATCGCCTGA
AAGAGCGGAAGGAGTCCTTCCTAAGATTCTTAAAAATCTTTTAAGTTCTAGAAAAGCCACTAAGAAAGAAATGAATTCAGTTACAG
ATCCAAGTCTTAAGTCGTGTTTAAATGGTAGACAACTTGCTTTTATACTTTGTGCCAATTCAATTTATGGATTTACGGGAGCTACGG
TGGGAAAATTGCCTTGCTTAGAAATATCGCAAAGTACTACGTCTTTCGGGCGAGAGATGATTGCGTTCTCGAAGAAATGCATTGA
GGAGACATTCTGCAAAAGTAAAGGATTTTCACACGATTCAGAAGTAATTTATGGTGATACAGACTCGGTAATGATAAATTTTCATGA
GACAGACATGAAAA***AAGCTT***GATCCGGCTGCTAACAAAGCCC

N. bombycis epsilon DNA pol. (in pRSETRNAi 2 [45]) 610 bp, G/C 30.7%, 15 bp and longer regions without adjacent G or C 65.4%

GGATCCTGAAAATGCACAAGATAAAGAAGATAATCATAAAAAGAACAAGTACAATGAAGATCCTACGAGGTATGAAGATATGACAG
ATTCTGAAAGATCAAAGTTATTAAAAAGTAAAATTTTTGAATATAGTAAAAAGAAATATAATTCTGCAACAATTCGTAAAACAGAAACA
CAAACTACAACTGTTTGCCAACGAGAAGTGCCGTTTTTTGTAAATGCAATTATTCAACTTCAAAAAAAAATAAATCATTGTGAATCT
GAACAAATATCGGCACTAAAAAATACAGTAGGATTGCCAAAAAAATCCGAGACTGAGCTTGATGTTTCATTATCAACGTATAAAAGC
ATATTAGAGTGGTTTCATGAGTATATTACTAAAAATAATTCAAGGTGGTACAGTATTGAGAGTGTTGGAATTGTCGGTAATCTCTGTA
CTAAACTTCAGAAAACATTAAAACAGAACATAGATAATATTGGACTTGTTCTGGATGTTGATACTAACTATATTTGGACCCTTTTACC
GGTTAAGTTTCCTAATATTTATAAATTCGAATCTGGGCAAGAAATTGTATTTATAAA***AAGCTT***GATCCGGCTGCTAACAAAGCCC

N. bombycis helicase (in pRSETRNAi 2 [45]), 475 bp, G/C 29.9%, 15 bp and longer regions without adjacent G or C 62.3%

GGATCCTAAATCTTTATAATCAGGGCGGAAATCATGACCCCATTGAGATACACAATGAGCTTCATCTATTACAAAATTACTTAATCTA
TTAATATTAAACTCTAAAGATTTATGGAATTGATCACTTTGAGCTATTAATTCAGGAGTAACATAAAAGATTTTACAAATCAATTCTTCA
GAATAAAGTAAATCATAAGCCAATCTCCTTTCTAAAGTAGATAGAGTAGAATTAATAGGCATAGCCAAGATATTCTTTTCTAATAAATT
AGTAATTTGATCTTGAACCAAAGAAAGTAAAGGACTTACTATGATAGTAATACCATTTTTAATTAAAGCAGGAATTTGATAACAAAGA
CTTTTACCCCCACCCGTAGGCATTAAAACAAAAACATCTTCATTTTGTAAATTAGCATGAATAATTTCCCTTTGATTTCTCCTAAAATC
TTT***AAGCTT***GATCCGGCTGCTAACAAAGCCC

N. bombycis topoisomerase II (in pRSETRNAi 2 [45]), 564 bp, G/C 29.3%, 15 bp and longer regions without adjacent G or C 64%

GGATCCTATTGAGATGCACAAGGAAGAAAACGTTTACGTACCCGAGTTGATTTTTGGTCAACTTCTCACTTCTTCTAATTATGATGA
TAAAGAAAAGAAAGTTACAGGAGGACGAAATGGATACGGTGCTAAACTTTGTAATATTTTTAGTAAAGAATTTATTGTGGAGACAGC
TGATTATTCAAAGAAAAAGATTTATAAACAAGTTTATAAAAAAAAATATGAGTATCACAGAACCACCTACTATTGAAGGTTATAGAGATA
AAGGATTTACGAAAATTACATTCAAACCAGATCTAAAAAGATTTAAAATGGAAAATTTGGATTCTGATATCGTATCTTTACTAAAGAAA
AGAGTTTATGATCTTAGTGCTACAGTTAAAAAGATAAAAGTTTATTTGAATGATGAACAAATAACGGTTTCTGGATTTAAAGATTATGT
AAAATTATATTTACCAGAAGATACTAAAATTATACATCAAGTTATAAATGATAGATGGGAATTAGGATTTACAGTTAGTGATGAACATTT
TCAAC***AAGCTT***GATCCGGCTGCTAACAAAGCCC

N. bombycis ligase (in pRSETRNAi 2 [45]), 532 bp, G/C 30.8%, 15 bp and longer regions without adjacent G or C 62.2%

GGATCCATCAAAATAAAGGCAATCAAAAATAAAAACGCAAATATTAACATTCTTAAAAGAAGATGAGGATGTATTTATGGGAATGTTG
CTAGATGAAGATAGTTCATTATTCTTATTACCACTTTCTTTCTTATTGGCTTTCCTTTTTCTTGTTGATAATTCTTGGAAACTCATTATT
TTCTTATTTTCTTTATCATAAGCCACTACTTCCCCATCCAAAATATAATCTTTATTATTTTTGGATTTAATTTTGATATCCGGGTACTTCT
CGGAATTATCTTCTAAATTCCTAGAAAATGTTCTAGAACAATTTTTAGAACTATGAATTTGGACCCGTTCTCCATCGTATTTGAACTC
ACAAGTAAATTTAGAGTTTTCAACTTTTTTATAAGCAGAAGATACGTCTTTAGAAGGAGTAGCCAACATTGGTTTAAGGGGGATACC
TGGAGTCATTGTGAAATTTATTAAAGAATTAATGCCTTCCTCTTGTAAAAATTTAACTAA***AAGCTT***GATCCGGCTGCTAACAAAGCC
C

Figure 2S. Sequences of five fragments of genes encoding *N. bombycis* DNA replication enzymes used for dsRNA synthesis. Regions without adjacent G or C are marked as grey boxes, cloning sites of *Bam*HI and *Hind*III restriction enzymes are in bold and italic, additional vector-derived sequences are underlined. Sequences of the control gene fragments with normal GC content used for dsRNA synthesis.
