# Supplementary material for: GC Content and Thermal Stability of Double-Stranded RNA: Fragments of Microsporidia Vairimorpha ceranae and Nosema bombycis AT-Rich Genes Are Sensitive to Standard Heat Treatment
Source: Int J Mol Sci. 2025 Oct 22;26(21):10270. doi: 10.3390/ijms262110270 (PMC12609917; doi:10.3390/ijms262110270)
Supplement: Supplementary file 1 [file ijms-26-10270-s001.zip › Figure S3. Sequences of V. ceranae fragments.pdf]

V. ceranae delta DNA pol. (in pRSETRNAi 2 [45]), 590 bp, G/C 31%, 15 bp and longer regions without adjacent G or C 55.3%

GGATCCATGCTATAAGAAGATCCTTCTCCGTGTTAAAACTTCTTACTATTGCACCGGGAATTGGCGCAGTTTCTTTTAAACAGAAAATTACC
TGCTCAATCTTGCTAGGATCATCTGAATAAGAAACAGTATTACCGATCTGTATAATTGGATCTTTTGTAGCTACTGGAAAAGATATTTTATCTA
AGCTAATACATTCTATGTCTATACTTAAAATTTTAAATTTAGGCAGCTTGACATATTCATTTTTAATTTCCAGTGGCACAACATCCACGTAAGAA
CAGGATATTACAAATGGATTCGATGATAAAACAATATATCGCTTAATTTTAAGATATTGCATTCCAACAATATTCATATCAACCATAAATCTTAAG
ACAAATGGAAAATTACTTTCGAATAATTTAAAGCGCACCAACCGATTTTTTATTAGAATTCCCGCTTCAAGAATTGGCTTTATTTGATAAAAAA
CTGATGGAGTATTAAAAGTTAATTTATAAAATAATGACTTAGATTCAGAATATCCATACAAAGGATATTTCATAGATGTACTGACATTTAA***AAGC***
TTGATCCGGCTGCTAACAAAGCCC

V. ceranae epsilon DNA pol. (in pRSETRNAi 2 [45]), 496 bp, G/C 26%, 15 bp and longer regions without adjacent G or C 78.4%

GGATCCGTGTGAAAAATATAAAAAGTCTTCATACAAAGTAATTGAAAATTTTCAATCTATTGTATGTCAGCTAGAATTTTCTTTTATGCTTA
ATGTATTGAAAAAATTTAAAGAATATTTTATTTTGTACAAAAACAATCTTGAAAATATTAAAAAAGAGCTAAAAGAAAATTATAGTTCAGAGA
ATTTAAAAAAGAGTAAATTAACATCTTCCATATACTGGGCACATGAATATCTCTACAATTCATTTTGTAAATATATTTATTATGAAGGCGCT
AGATGGTATTCAACAGAAATGACAATAATATTGGATAGTATCGCTACTGAGATTATAAAAAATTGTACTACTTTGTTAAACAATTTTTCTCT
TCCTTTGATTGTAAATTCAGAAAGTATTTGGACATTGTTACCTTTTACATTCCCTCAAGAAGTTGAATTTAAATCAGGTAAAAAATTTAATTT
TCTTGA***AAGCTT***GATCCGGCTGCTAACAAAGCCC

V. ceranae helicase (in pRSETRNAi 2 [45]), 594 bp, G/C 33. 4%, 15 bp and longer regions without adjacent G or C 56.2%

GGATCCCTATACAGAGGTTATGACACCACAACTCGATATTTTACGAAATGTTTTTAAGTTAGAAAAATTTCGTACGAATCAGGAGTCTATTAT
AAAATCAATTCTAGAAAAAAAAGATGTATTTGTATTAATGCCTACTGGTGGCGGAAAATCTTTATGTTATCAAATACCCGCTCTAATAGACAAT
GGGGTTACTATTATTATCAGCCCACTACTTTCACTGGTACATGATCAAATATCTAATTTATTAAATAACAACATTTTAGCCTTACCTTTTAATTC
TACACTAAATGCATCAGAAAGGCGTATGGTTCTAGAAAATATGACTTTAGGGGTAGTTAAAATGTTTTATGTAACTCCTGAATCCTTATGTGC
CAATTATAATTTAGAAAGTAAACTTAAAGAACTACTTCGTATGAATAAACTTAGTAGGTTTGTTGTTGATGAAGCACATTGTGTCAGTCAGTG
GGGGCATGACTTTAGGCCGGATTATATTGAAATGAAAAAAATCAAAGAAATGTATCCATCAGTGCCTATAGTAGCACTTACAGCTACCGCTA
CACCAA***AAGCTT***GATCCGGCTGCTAACAAAGCCC

V. ceranae topoisomerase II (in pRSETRNAi 2 [45]), 641 bp, G/C 32.5%, 15 bp and longer regions without adjacent G or C 46.7%

GGATCCAGAAGATGCAGTTGTAGTGCATGAAATAATAAATGAAAGGTGGGAACTTGCATTTACAACTAGTGAAGAACAATTTCAGCAAGTT
TCATTCGTTAATAGTATTTCTACATCAAAAGGTGGGTCACATGTAAATCACGTTGTAGAACAGCTTACTGATCCAATAATAGAAGCTCTTAAA
AAAAAGAAAATTAGTAATGTAAAACCATTTCAGGTAAAATCATCTATGTTTTTATTTATAAATTCATTAATTGATAATCCAGCATTTGATTCACAG
ACTAAAGAAAATTTAACTTTACGTGTGGGAGCTTTTGGTTCTAAATGTGAACCTTTAAAAGATTTTATTGATAAAGTCATAAAAAATACAGAA
GTAGTTACGAAAATAGCAGATTTTGTAAAAGCGAAAGAAGATGCTGCATTAAAGAAAACTGATGGTAAGAAAAAAGGACGAATAATTATTGA
TAAACTTGAAGATGCAAAATGGGCGGGCACTGCTAAATCTAATGAATGTACATTGTATCTAACAGAAGGAGATTCTGCTAAAACGATGGTAA
TGTCAGGAAGGTCTATTGTAGGAACAGAAAGACTAGGTGTGTATCCTTTAAGAGGT***AAGCTT***GATCCGGCTGCTAACAAAGCCC

V. ceranae ligase (in pRSETRNAi 2 [45]), 549 bp, G/C 29.3%, 15 bp and longer regions without adjacent G or C 62.7%

GGATCCGGAATAAAATCTAGAATTTACAAAATTCGTAAAATGTTGGATCTTATCTCTTCATGCACTCCTATAGAAATAAAGTTTTTATTCAGAT
TATTTGAAGAAAACTTAAAAGTAAAATTTGCCTTGAAAACTGTACTGGCAGCATTTGCTAAATTGTATGATAGTGCTTACATTGATAAAATAAA
AGAGGCATATAATAGACGACCAGATATAGAACAACTTGTTATAGAAATTCTTACTAACGGCATATCAACAGTAGATACAAATTTCAATATTGAA
CCTGGTATTCCACTTAAACCGATGTTAGCACAACCCACCAAGAATATTTCTACTGCATTTAAGCGTGTCGAAAATAAAAAATTTACATGTGAA
AATAAATACGACGGTGAAAGAATACAAATACACCGACACAACAACCAAATGACATTGTATTCAAGAAATTTAGAGAATACAACAGATAAATAT
TTTGATATAATTATTAAATCAAACACTGATAAAGATTTTGTAATAGACGGAG***AAGCTT***GATCCGGCTGCTAACAAAGCCC

Figure 3S. Sequences of five fragments of genes encoding *V. ceranae* DNA replication enzymes used for dsRNA synthesis. Regions without adjacent G or C are marked as grey boxes, cloning sites of *Bam*HI and *Hind*III restriction enzymes are in bold and italic, additional vector-derived sequences are underlined.
